# Developmental Transcriptome for a Facultatively Eusocial Bee, *Megalopta genalis*

**DOI:** 10.1534/g3.115.021261

**Published:** 2015-08-14

**Authors:** Beryl M. Jones, William T. Wcislo, Gene E. Robinson

**Affiliations:** * Program in Ecology, Evolution, and Conservation Biology, University of Illinois, Urbana, Illinois 61801; † Smithsonian Tropical Research Institute, Panama City, Panama 20521-9100; ‡ Department of Entomology, University of Illinois, Urbana, Illinois 61801; § Carl R. Woese Institute for Genomic Biology, University of Illinois, Urbana, Illinois 61801; ** Neuroscience Program, University of Illinois, Urbana, Illinois 61801

**Keywords:** development, eusociality, social evolution, phenotypic plasticity, transcriptomics

## Abstract

Transcriptomes provide excellent foundational resources for mechanistic and evolutionary analyses of complex traits. We present a developmental transcriptome for the facultatively eusocial bee *Megalopta genalis*, which represents a potential transition point in the evolution of eusociality. A *de novo* transcriptome assembly of *Megalopta genalis* was generated using paired-end Illumina sequencing and the Trinity assembler. Males and females of all life stages were aligned to this transcriptome for analysis of gene expression profiles throughout development. Gene Ontology analysis indicates that stage-specific genes are involved in ion transport, cell–cell signaling, and metabolism. A number of distinct biological processes are upregulated in each life stage, and transitions between life stages involve shifts in dominant functional processes, including shifts from transcriptional regulation in embryos to metabolism in larvae, and increased lipid metabolism in adults. We expect that this transcriptome will provide a useful resource for future analyses to better understand the molecular basis of the evolution of eusociality and, more generally, phenotypic plasticity.

Transcriptomes provide excellent foundational resources for mechanistic and evolutionary analyses of complex traits in both model and nonmodel organisms. For example, in human disease research, variation in cell- or tissue-specific gene expression has implications for personalized medicine, and transcriptomics has become an attractive approach for cancer diagnosis and therapy choice (Parker *et al.* 2009; Rosenwald *et al.* 2002). Evolutionary biologists studying natural populations have increasingly used transcriptomics to bridge the gap between environment and phenotype by revealing context-specific gene expression and the function of novel transcripts and genes (Alvarez *et al.* 2015). Understanding the extent of gene expression variation can address how responsive a population may be to novel environmental conditions (Oleksiak *et al.* 2002; Whitehead and Crawford 2006), and variation in expression can itself be a target of selection (Oleksiak *et al.* 2002; Whitehead 2012).

We present a developmental transcriptome for the facultatively eusocial halictid bee *Megalopta genalis*. Our goal is to provide a tool that will enable *M. genalis* to be used in comparative transcriptomic analyses to better understand the evolution of eusociality, one of the most extreme forms of animal developmental phenotypic plasticity. Eusociality evolved independently at least 24 times, nine or more within the hymenopteran insects (Bourke 2011; Cameron and Mardulyn 2001; Cardinal *et al.* 2010; Hines *et al.* 2007), but absence of extant ancestral lineages prohibits the direct study of eusocial origins.

A promising approach to studying the origins of eusociality is to study incipiently social species across different lineages in a comparative context (Kocher and Paxton 2014). Particular bee groups are especially well-suited for this task due to the variation in the expression of sociality within and among species. One strikingly diverse group of bees is the family Halictidae, which is a cosmopolitan taxon comprising greater than 4000 species with behavior that ranges from solitary to eusocial (Michener 1990). Within one subfamily, the Halictinae, at least three independent origins of eusociality have been identified (Danforth 2002), all of which occurred approximately 20–22 million years ago (Brady *et al.* 2006). Additionally, there may have been a number of reversions to solitary life among the halictids, suggesting this group is especially flexible and able to transition between solitary and social states (Kocher and Paxton 2014; Wcislo and Danforth 1997). Finally, the subfamily Halictinae includes species that are facultatively social, in which females of the same population can produce either solitary or eusocial nests (Packer 1990; Cronin and Hirata 2003; Eickwort *et al.* 1996; reviewed in Kocher and Paxton 2014).

One such facultatively eusocial species is *Megalopta genalis*, a Neotropical bee common in the rainforests of the Americas and especially well-studied on Barro Colorado Island in Panama (Wcislo *et al.* 2004). Foundress females of this species can produce either solitary nests, with only male offspring in the first brood, or small eusocial nests with at least one daughter worker in the first generation (Smith *et al.* 2003; Wcislo *et al.* 2004; Kapheim *et al.* 2012b). Both solitary and eusocial nests may produce a mix of dispersing males and females in later generations, and differences in sex ratio are not due to mating status of females because all reproductive females are mated (Kapheim *et al.* 2012a). Instead, flexibility in nest sociality is a result of larval and adult environmental influences (Smith *et al.* 2003; Kapheim *et al.* 2012b), and may represent a transition point in the evolution of eusocial insects. If the phenotypic flexibility present in *M. genalis* captures an evolutionary transition in social behavior, then understanding the mechanisms of this flexibility may open a window into the origins of eusociality. However, *M. genalis* is currently limited as a model for social transitions due to the lack of resources for studies of gene expression or genetic underpinnings of social flexibility.

As the first step toward using transcriptomic analyses of *M. genalis* to better understand the role of developmental phenotypic plasticity in the origins of eusociality, we sequenced and assembled a developmental transcriptome. We used this transcriptome to conduct a preliminary survey of the extent of plasticity in gene expression in *M. genalis* across development and identified molecular pathways with highly plastic gene expression. In the future, we expect this reference transcriptome to be useful in studies of gene expression in *M. genalis* as well as in a comparative framework with other social insects in studying the developmental origins of eusociality.

## Materials and Methods

### Sample collection and tissue preparation

**Figure 1:**
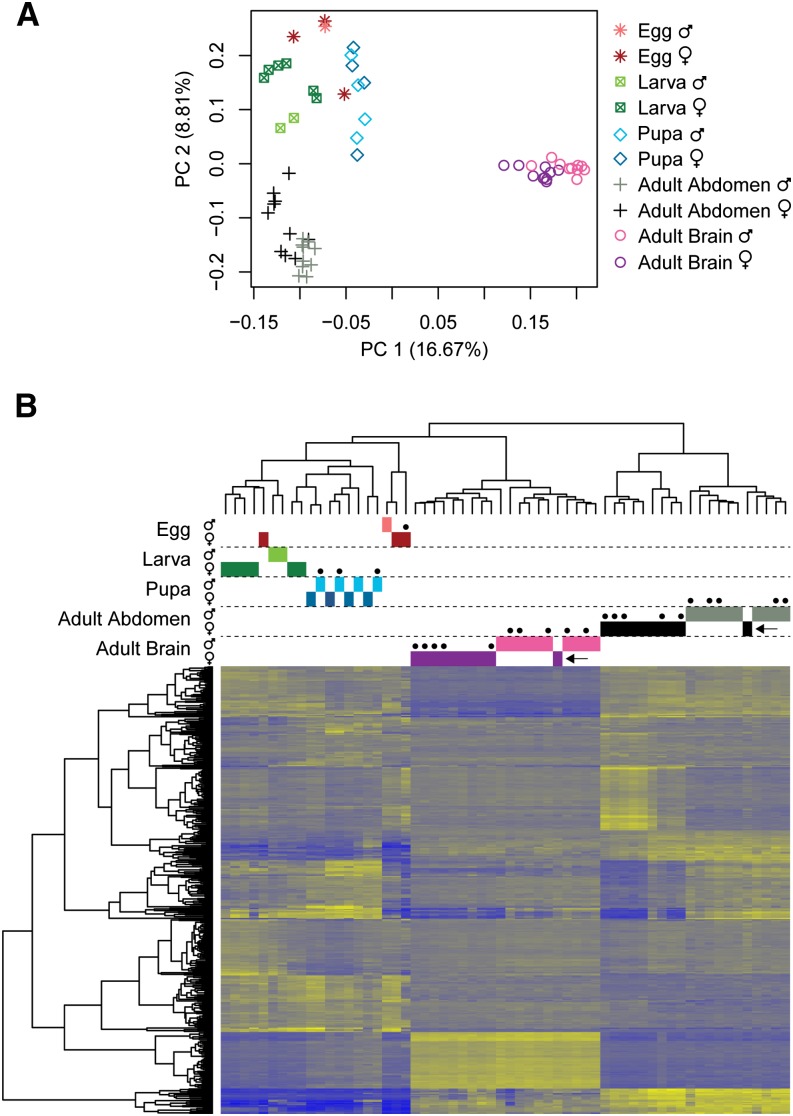
Clustering of individuals based on overall patterns of gene expression. (A) PC plot for first two principal components and (B) heatmap and clustering of 400 genes with the most variable gene expression across samples (100 most variable genes from each of the first four principal components). Each row of the heatmap represents a single gene, and genes are clustered based on expression similarity. Dots above heatmap indicate individuals reared in the laboratory or placed in observation nests prior to collection (as opposed to collected from natural nests in the field). Blue and yellow colors in the heatmap correspond to low or high relative gene expression, respectively. Arrows refer to individual discussed in *Principal component analysis* section of *Results and Discussion*.

Collections were made on Barro Colorado Island (BCI) in the Republic of Panama, a 1500-hectare island in Lake Gatun formed during construction of the Panama Canal. *Megalopta* species are nocturnal insects active during the dry season, with densities of approximately 5×10^−3^ nests per square meter (Wolda and Roubik 1986; Wcislo *et al.* 2004). Some individuals were collected from natural nests and then placed into liquid nitrogen. Other individuals were collected as larvae or pupae from natural nests and then reared through the adult stage at ambient temperature in an outdoor enclosure prior to liquid nitrogen freezing. A subset of females was placed into observation nests (as described in Kapheim *et al.* 2011) after eclosion and collected after nest construction and egg laying had begun (rearing and age information where known is provided in Supporting Information, File S3). Differential effects of rearing condition were not apparent from clustering of samples based on gene expression variation (Figure 1B). A total of four eggs, eight larvae, eight pupae, and 20 adults were used for sequencing. Pupal and adult samples were balanced for sex, and the sexes of earlier life stages were determined postsequencing as explained below (see *Sex determination of preadult stages*).

For adult samples, whole brains were dissected from the head capsule on wet ice following 16-hr incubation at −20° in RNA-later ICE (Life Technologies), with frons and cuticle around postocciput removed prior to incubation. Abdomens were similarly incubated in RNA-later ICE prior to removal of gut tissue and Dufour’s glands. Preadult life stages were not dissected. Many tissue types were included for sequencing to maximize the number of transcripts captured for transcriptome assembly; however, a caveat of the data is that tissue- or cell-type resolution is lost. Total RNA was extracted using QIAGEN RNeasy Mini Kits [treated with DNase (QIAGEN) to remove genomic DNA] and quality was confirmed with Bioanalyzer RNA Pico chips (Agilent) prior to library preparation.

### Library preparation for RNA-sequencing (RNA-seq)

Poly-A RNA was enriched from 0.6 to 1.0 µg total RNA using NEXTflex Poly(A) Beads from Bioo Scientific. Strand-specific cDNA libraries were prepared using the Bioo Scientific NEXTflex Directional RNA-Seq Kit (dUTP Based) for Illumina following manufacturer instructions with a 12-min fragmentation time and 15 PCR cycles. Libraries were barcoded with Bioo Scientific adaptors so they could be pooled for sequencing. For the 20 adult samples, two libraries were created per individual: one for whole brain and one for abdominal tissue. In total, 60 libraries were created for the 40 individuals in the study. Library concentrations were quantified using a Qubit dsDNA High Sensitivity Assay Kit (Life Technologies), and library size was assessed using a Bioanalyzer High Sensitivity DNA chip (Agilent). Libraries were pooled into four groups at equal concentration and diluted to final pooled concentrations of 10 nM. Library pools were quantified using the Illumina-compatible kit and KAPA standards for real-time PCR by the W. M. Keck Center for Comparative and Functional Genomics at the Roy J. Carver Biotechnology Center (University of Illinois).

### Sequencing and preassembly read processing

Paired-end sequencing was performed on an Illumina HiSeq2000 at the W. M. Keck Center. Fifteen libraries were sequenced on each of four lanes, resulting in over 1.6 billion reads (averaging 27.12 million per library). Quality was assessed using FASTQC (v. 0.10.1), and read trimming was performed with Trimmomatic (v. 0.32) to remove low-quality reads and remaining adapter sequences. Ninety-eight percent of reads passed quality and adapter trimming across all samples. For assembly, reads were concatenated into single files prior to running a digital normalization to a maximum coverage of 50×. This normalization reduced the number of input reads by nearly 90%, dramatically reducing computer processing time, and was expected to result in little to no loss of transcript information (Brown *et al.* 2012).

### Trinity assembly

An initial *de novo* assembly was performed using Trinity (v20140413) and included all 60 libraries. This assembly resulted in nearly 197,000 genes and 256,000 transcripts, a highly unrealistic number of transcripts given what we know from other bee genomes (an average of 13,616 genes for 10 bee genomes sequenced) (Kapheim *et al.* 2015). Mapping these transcripts to the closest related genome available, that of *Lasioglossum albipes* (Kocher *et al.* 2013), revealed that many transcripts and genes were mapping to the same loci. To reduce the complexity of reads for assembly, we took advantage of a set of five related individuals within our sample group (Figure S1). RNA from these individuals was used to make nine libraries, which were sequenced and resulted in a combined total of 142 million reads. As before, digital normalization was applied to 50× using Jellyfish. Trinity (v20140413) was again used for assembly and, as expected, the number of genes and transcripts was reduced to 75,206 genes and 102,303 transcripts. Additionally, the contig N50 and other measures of assembly continuity and quality improved (contig N50: increased from 1056 to 2057 bp; mean contig length: increased from 658.19 to 868.98 bp). Finally, the percentage of reads mapping (methods described in *Read alignment and abundance estimation*) to this assembly was higher than the previous assembly (84% *vs.* 82% average per sample). This assembly was therefore used for the remaining analyses presented. Assembly statistics are reported in Table 1, where gene-level metrics are based on the longest isoform per gene.

**Table 1:** Summary of assembly statistics

Category	Number				Total Number	Mean Length (bp)	N50 (bp)	Total Nucleotides
	200–499 bp	500–999 bp	1–1999 bp	≥2 kbp				
Transcripts	51,469	15,504	12,567	22,763	**102,303**	1390.39	3351	**142,241,080**
Genes	48,270	11,958	6728	8250	**75,206**	868.98	2057	**65,352,587**

Assembled transcripts were screened against the NCBI nonredundant (nr) database using BLASTX with an e-value threshold of 1e-5. Of the 102,303 transcripts, 37.52% had a significant hit to the nr database; 96.61% of these hits were to insects, and 95.34% of hits were to hymenopteran species. Although nr is one of the most complete databases of sequence information available to the public, it does not yet contain information from five bee genomes that have been recently sequenced (Kapheim *et al.* 2015). Using a BLASTN against a custom database consisting of all 10 bee genomes that have been sequenced to date (Figure S2), 55.20% of assembled transcripts (and 45.71% of genes) mapped to at least one location in at least one of the 10 bee genomes with an e-value threshold of 1e-5.

Completeness of the assembly was assessed using two sets of information from the 10 bee genomes. The first comparison set included 5855 single copy orthologs across all 10 genomes (Kapheim *et al.* 2015). All assembled transcripts were used as queries in a BLASTN (maximum e-value of 10e-3) against this set of orthologous genes. Similarly, all transcripts were mapped against each of the 10 bee genomes, and the percentage of unique genes with a transcriptome hit is shown in Figure S2 (range: 37.17–84.56%).

### Read alignment and abundance estimation

Quality- and adapter-trimmed reads for all 60 libraries were aligned to the transcriptome using the align_and_estimate_abundance.pl script in the Trinity r20140413 toolkit, which uses Bowtie (version 0.12.7) for alignment and RSEM (version 1.2.10) for estimating transcript abundance. An average of 84% of reads per sample (min: 74.14%; max: 87.95%) reported at least one alignment to the transcriptome. For all downstream analyses, only read counts at the putative gene level (not transcript level) were used.

### Sex determination of preadult stages

The Haplotype Caller within the Genome Analysis Toolkit (GATK) was used to predict sex for the preadult stages. Because males are haploid, the number of confidently called SNPs for a male should be small compared to the number of SNPs found in diploid females. As a proof of concept, 12 adult individuals (of known sex) were run through Haplotype Caller. Female individuals (n = 6) had 23,857 ± 3428 SNPs called (suggestive of heterozygous loci) while males (n = 6) had only 3509 ± 1564 SNPs using the same filtering criteria (these SNPs could be the result of paralogous gene sequences, sequencing errors, or assembly artifacts). The sexes of all eggs and larvae were assigned based on the number of SNPs called using Haplotype Caller, with all predicted females having greater than 12,500 SNPs and all males having less than 3800 called SNPs. Three of the four eggs and six of the eight larvae were predicted to be female based on these criteria.

### Principal component analysis

Broad clustering of gene expression profiles for all individuals was conducted using a Principal Component Analysis (PCA) of TMM-normalized (Robinson and Oshlack 2010) FPKM values obtained using RSEM within the Trinity script abundance_estimates_to_matrix.pl. The PtR.pl script packaged with Trinity, which utilizes a number of plotting functions in R, was used to produce the plot and heatmap presented in Figure 1. Genes with less than 10 FPKM counts across the 60 libraries were excluded prior to clustering, and data were log2 transformed prior to PCA. The most variable 100 genes (based on extreme eigenvalues) for each of the first four principal components (400 genes total) are shown in the heatmap and clustering dendrogram. This analysis provides a visualization of gene expression based on the most variably expressed genes across all samples.

### Developmental dynamics of gene expression

**Figure 2:**
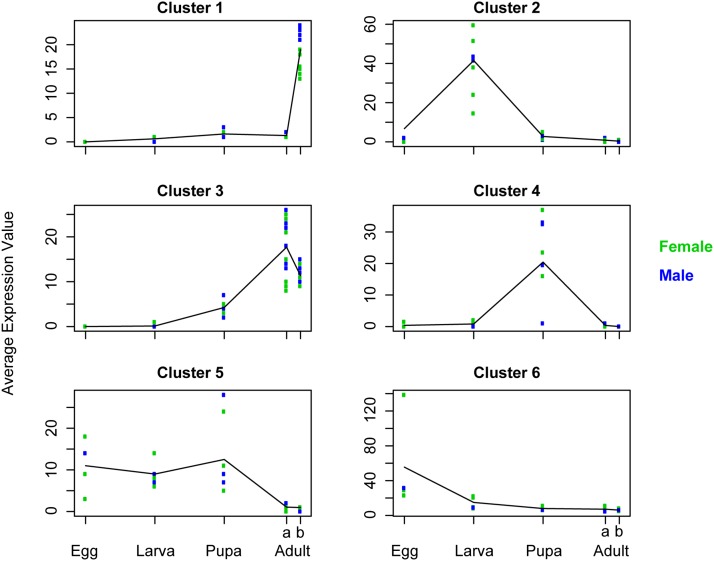
Clusters of genes with similar gene expression patterns throughout development. Each symbol represents the median expression for all genes within the cluster for one individual, and lines connect the average expression value across individuals for each life stage. Along the x-axis, “a” and “b” refer to adult abdominal and brain tissues, respectively.

We conducted a preliminary survey of gene expression changes throughout development using the R package maSigPro, which uses a GLM regression approach to find clusters of genes significantly differentiated through time (*i.e.*, across life stages; a linear step-up Benjamini-Hochberg false discovery rate procedure was used, with corrected *P* < 0.05 for all genes) (Conesa *et al.* 2006). The 75,206 *M. genalis* assembled genes were filtered to include only those with least 1 count per million (CPM) in at least two samples, resulting in 22,315 genes for analysis. To avoid inappropriate grouping of potentially distinct groups of genes, we initially used the maximum number (n = 9) of clusters for maSigPro, followed by paring down to six clusters based on similar expression patterns of three pairs of clusters (the original nine clusters are shown in Figure S3). A design matrix was formed that described the life stage and tissue (egg, larva, pupa, adult-abdomen, adult-brain) of each individual. For each cluster of genes, the median expression value of those genes for each individual is calculated, and this median for each individual is then averaged across samples for visualization in Figure 2. The analysis was repeated excluding males, and results looked very similar (Figure S4).

For each life stage, we identified genes more highly expressed in that life stage (for adults considering abdomens and brains separately) than in any other life stage using differential gene expression analysis with edgeR (Robinson *et al.* 2010). The filtered set of 22,315 genes with CPM ≥1 in at least two samples was used for analysis. Count data (obtained from RSEM as discussed above) were normalized by library size and library composition (TMM) in edgeR. Dispersion was estimated across samples using the estimateGLMTagwiseDisp function in R (following estimateGLMTrendedDisp) such that estimates were squeezed toward the trended dispersion values with a prior degrees of freedom value of 20 (McCarthy *et al.* 2012). Raw *P* values from each test were commonly corrected using the p.adjust function in R using the Benjamini-Hochberg ("FDR") method. Genes that were more highly expressed in one life stage compared to all other stages (using an FDR-corrected *P* of 0.05 as the significance cutoff) were functionally annotated using PANTHER, and statistical overrepresentation tests were conducted on those lists relative to the reference set of 22,315 genes used in the edgeR analysis.

To assess changes associated with transitions between life stages, we used PANTHER overrepresentation tests on differential expression lists obtained from edgeR such that each life stage was compared with life stages directly preceding or following that stage (the pupal stage was compared with the adult brain and adult abdomen samples separately). For example, genes that were more highly expressed in larvae compared with eggs were compared to the reference 22,315 genes to test for overrepresentation of GO-Slim and PANTHER protein categories as described below.

### Functional annotation of genes with PANTHER

TransDecoder (r20131110, packaged with Trinity r20140413) was used to identify candidate coding regions within assembled *M. genalis* transcripts. The predicted peptides for the 22,315 genes that passed the minimum expression threshold were used as input to PANTHER (pantherScore1.03, library version 9.0) to identify protein family domains. A statistical overrepresentation test with Bonferroni correction for multiple testing was used to identify biological processes and protein classes that were overrepresented in the overexpressed gene lists for each life stage relative to the reference set of 22,315 genes. These analyses were conducted using the Gene List Analysis tools available on the PANTHER website (pantherdb.org) (Mi *et al.* 2013).

### Data availability

File S1 contains enriched terms from PANTHER analysis of *M. genalis* gene clusters. File S2 lists enriched terms from PANTHER analysis of genes overexpressed in each life stage. File S3 provides rearing and age information for sequenced individuals. Sequencing reads used for transcriptome assembly and differential expression analysis have been deposited in the short read archive (SRA, NCBI) under the accession number SRP057750.

## Results and Discussion

### Reference transcriptome assembly statistics

To reduce the complexity of reads for assembly, we took advantage of a set of five related individuals within our sample group (Figure S1). RNA from these individuals was used to make nine libraries (RNA from brains and abdomens in separate libraries for adults), which were sequenced and resulted in a combined total of 142 million reads; 75,206 Trinity components (hereafter referred to as genes) were assembled from these reads, yielding a contig N50 of 2057 bp. Additional assembly statistics are presented in Table 1. Assembly completeness was assessed in two ways using data from genome sequences of 10 bee species (Kapheim *et al.* 2015): (1) we determined the presence or absence of 5855 single copy orthologs identified as common to all 10 bees and (2) we compared our list of putative genes in *M. genalis* with the genes identified in each of the 10 sequenced bee genomes. Ninety-seven percent of genes within the orthologous gene set were found in the *M. genalis* transcriptome, indicating that sequencing depth and assembly parameters were sufficient to capture nearly all highly conserved transcripts. The two species with the highest percentage of genes with sequence homology to transcripts in the transcriptome are *Lasioglossum albipes* (75.33%), the species most closely related to *Megalopta genalis*, and *Apis mellifera* (84.56%), the species with the most thoroughly annotated genome (Weinstock *et al.* 2006; Elsik *et al.* 2014). The phylogeny of the 10 bee species used for comparison and the percentage of genes from each of the species with a homologous *M. genalis* contig are shown in Figure S2.

### Read alignment and abundance estimation

RNA-seq libraries sequenced from 40 individuals spanning all life stages and both sexes were aligned to the reference assembly using Bowtie, and abundance estimation was conducted using RSEM. An average of 84% of quality-trimmed reads mapped to the assembly from each library (range: 74.14–87.95%); 22,315 genes (approximately 30% of total genes) had an expression value of at least 1 CPM in at least two samples. This stringent reads-mapping cutoff was used to give high confidence that the genes in this set are not sequencing artifacts or a result of assembly errors, and the 22,315 genes above the 1 CPM cutoff were used for all differential expression and functional analyses. This number is similar to that found in a *de novo* assembly of the paper wasp *Polistes canadensis* (26,284 isogroups) (Ferreira *et al.* 2013), but much lower than the number of transcripts (358,709) analyzed in the *de novo* assembly of the small carpenter bee, *Ceratina calcarata* (Rehan *et al.* 2014).

### Survey of gene expression

#### Principal component analysis:

PCA was conducted to identify broad patterns of gene expression and genes that best discriminate sex and life stage groups. The first four principal components (PCs) explained a total of 35.7% of the variance in expression (16.7%, 8.8%, 6.3%, and 3.9%, respectively). Clustering of samples based on the first two PCs is shown in Figure 1A, and a heatmap of 400 genes with highly variable expression (100 most extreme genes from each of the first four principal components) is shown in Figure 1B.

As evident from the heatmap in Figure 1B, the most variable genes separated adult tissues from preadult life stages. Within adults, males and females clustered separately, with one exception (highlighted with arrows in Figure 1B) being a single female clustering with males in terms of both brain and abdominal gene expression patterns. This female is unique in that she was newly eclosed when collected, while all other females were collected from nests and are mature adults (potentially months old; File S3). Males only remain in the natal nest for a few days prior to dispersal, thus all males in this study are very likely less than a few days old. Clustering of a very young female with the males suggests that young adults of *M. genalis* may be similar in gene expression regardless of sex, which is in contrast with the obligately eusocial honey bee, which has drastic differences in brain gene expression between sexes even at 1 d old (Zayed *et al.* 2011). Intriguingly, males and females of *M. genalis* are much more similar in terms of their size and gross morphology than are females and drones of *A. mellifera*, and perhaps the gene expression patterns in the two species reflect the respective levels of morphological differentiation found between sexes. Because the focus of this study was to collect diverse samples for the reference transcriptome, constraints on collecting age-matched males for comparison with adult females prevent a powered analysis of sex differences in gene expression in the adult stage.

For preadult stages, sex was much less predictive for sample clustering and all pupae were completely intermixed with respect to sex. Egg and larval stages also showed little differentiation based on sex, although sample sizes for males of these stages were small (only one male egg and two male larvae). One female egg clustered more closely with larvae than the other eggs. Because the time of egg laying is unknown for these individuals, one possible explanation is that this egg was close to hatching into the first larval instar. The lack of differentiation between sexes in these early life stages may also reflect differences in the specific developmental time points of the individuals. It is interesting to note that despite the variation included within each life stage (*e.g.*, multiple embryonic stages, different larval instars) and the very small sample sizes, life stages were still strongly differentiated in terms of gene expression.

#### Developmental dynamics of gene expression:

We utilized three separate analyses: (1) a GLM regression approach to find clusters of genes differentiated through time (maSigPro) (Conesa *et al.* 2006); (2) edgeR to find genes most highly expressed in each life stage relative to all others; and (3) edgeR to find genes differentially expressed in each transition between life stages (Robinson *et al.* 2010).

Genes were annotated for biological functions using the PANTHER database, and statistical overrepresentation tests were conducted on each gene list relative to the reference set of 22,315 genes with CPM ≥1 in at least two samples (Mi *et al.* 2013). To describe major differences among genes in each list, we identified the top unique overrepresented biological processes and PANTHER protein classes (Bonferroni-corrected *P* value, all *P* < 0.05) for each list. All significantly overrepresented and underrepresented GO-Slim Biological Processes and PANTHER Protein Classes for each analysis are listed in File S1 and File S2. Results from the three analyses were similar, adding confidence to the signal and providing a broad view of gene expression during each developmental stage of *M. genalis*.

The embryonic stage was dominated by signatures of transcription and DNA binding (Table 3), similar to genes in Cluster 6 (Figure 2, Table 2). This likely reflects the extensive pattern formation and regional specification that occurs during the embryonic stage, with transcription regulation factors such as Wnt establishing body axis patterning and cell fate during the embryonic stage (Cadigan and Nusse 1997). Cluster 6 was also enriched for a number of biological processes and protein classes related to RNA processing and RNA metabolism (Table 2). These genes may be responsible for the rapid differentiation of cell types during the embryonic stage of insects (Shields *et al.* 1975).

**Table 2:** Significantly overrepresented biological processes and PANTHER protein classes for *M. genalis* gene clusters

Cluster	# of Genes	GO-Slim Biological Process	PANTHER Protein Class
**1**	870	Ion transport, localization, cell–cell signaling, steroid metabolic process	Transporter, oxygenase
**2**	260	Proteolysis, metabolic process, protein folding, lipid metabolic process	Serine protease, chaperonin, hydrolase, storage protein
**3**	63	None	DNA photolyase
**4**	160	Unclassified	Unclassified
**5**	131	Cellular process, developmental process, cellular component movement, cell adhesion	Receptor, cell adhesion molecule, cadherin, tubulin
**6**	114	Nucleobase-containing compound metabolic process, mRNA processing, RNA metabolic process, RNA splicing	Nucleic acid binding, RNA binding protein, mRNA processing factor, mRNA splicing factor

The top four unique overrepresented terms (all Bonferroni-corrected *P* < 0.05) are shown corresponding to gene clusters shown in Figure 2.

**Figure 3:**
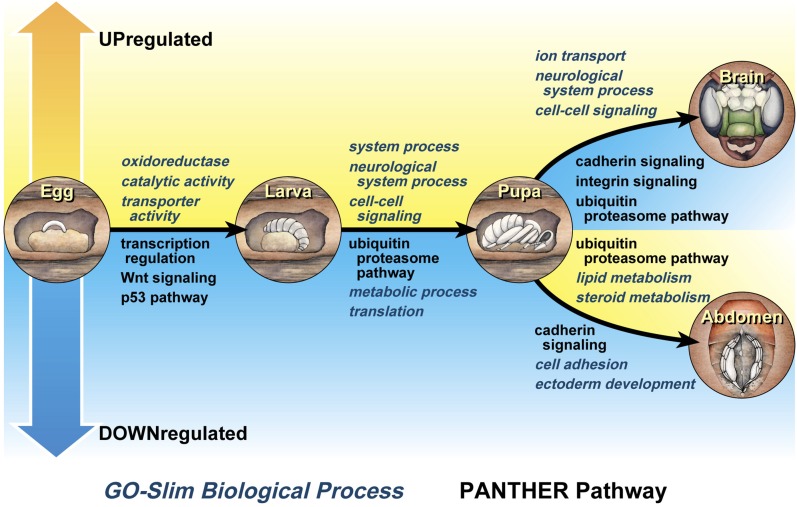
Functional annotation of genes showing differences in expression associated with pairwise transitions between life stages. Terms above transition arrows indicate genes that are more highly expressed in the life stage to the right of the arrow, while terms below the arrow indicate genes that are more highly expressed in the life stage to the left of the transition arrow. Terms in black are PANTHER Pathways, while blue italicized terms are GO-Slim Biological Processes. All terms listed are statistically overrepresented with a Bonferroni-corrected *P*<0.05. Artistic renderings of different life stages and tissue types are not representative of every sample included in the analysis, and only represent one particular life stage, sex, or tissue. Drawings by Julie Himes.

In transitioning to the larval stage, an increase in many metabolic functions and a decrease in transcriptional regulation were observed (Figure 3). High expression of genes enriched for metabolic processes may contribute to the rapid growth through the larval instars as individuals consume pollen provisions in their cells. This result is similar to what has previously been found comparing gene expression between larval and adult ants of *Camponotus festinatus*, with protein metabolism genes highly expressed during the larval stage (Goodisman *et al.* 2005). Elevated expression of metabolic and storage protein genes has also been reported in larvae of the bumble bee, *Bombus terrestris* (Colgan *et al.* 2011).

Larvae also showed the highest expression of genes involved in many enzymatic functions, including dehydrogenase and hydrolase protein classes (Table 3). This again highlights the turnover of metabolites during the larval growth stages of this insect. Finally, the ubiquitin proteasome pathway is more active in the larval stage compared with the pupal stage, reminiscent of the caste-specific expression of ubiquitin-related genes in larvae of the honey bee (Chen *et al.* 2012; Barchuk *et al.* 2007; Humann and Hartfelder 2011).

**Table 3:** Overrepresented biological processes and PANTHER protein classes for overexpressed genes in each life stage

Life Stage	# of Genes	GO-Slim Biological Process	PANTHER Protein Class
**Egg**	837	Nucleobase-containing compound metabolic process, DNA-dependent transcription, transcription from RNA polymerase II promoter	DNA binding protein, transcription factor, nucleic acid binding
**Larva**	717	Metabolic process, primary metabolic process, lipid metabolic process	Oxidoreductase, dehydrogenase, hydrolase
**Pupa**	793	Unclassified	Structural protein, unclassified
**Adult abdomen**	2392	Lipid metabolic process, fatty acid metabolic process, steroid metabolic process	Oxidoreductase, oxygenase, acyltransferase
**Adult brain**	4924	Neurological system process, system process, cell–cell signaling	Ion channel, ligand-gated ion channel, acetylcholine receptor

The number of genes significantly overexpressed in each life stage produced from pairwise comparisons of gene expression, as well as the three GO-Slim biological processes and PANTHER protein classes most highly overrepresented for each gene list (all Bonferroni-corrected *P* < 0.05).

Genes more highly expressed in the pupal stage relative to other stages were largely unclassified based on conserved protein domains (Table 3). Because the PANTHER protein database currently includes only two insects (*Drosophila melanogaster* and *Anopheles gambiae*), particular protein families important for insect metamorphosis may be underrepresented in PANTHER. Further, bee-specific protein families are absent from the database. However, structural proteins were enriched in the pupal stage, highlighting the extensive physical rearrangements of tissues occurring during metamorphosis. Relative to the larval stage, pupae also showed an increase in neurological system process genes and genes involved in cell–cell signaling, perhaps related to the reorganization of nervous tissues during the pupal stage (Technau and Heisenberg 1982; White and Kankel 1978).

Cluster 5 contained 131 genes expressed throughout egg, larval, and pupal stages but lowly expressed in adults. This cluster was enriched for genes involved in cellular and developmental processes, as well as cell adhesion (Table 2). This pattern of expression for adhesion genes has also been documented in *Drosophila melanogaster*, with relatively little expression in adults, but expression throughout earlier life stages (Arbeitman *et al.* 2002). Cluster 5 was also enriched for the cytoskeletal regulation by Rho GTPase pathway (*P* = 0.00112). The Rho family of GTPases is known to regulate a number of cellular functions important for cell shape, motility, and adhesion, as well as progression through the cell cycle (Narumiya and Morii 1993; Ridley 1995). The expression pattern of these genes as seen in Figure 2 suggests that factors influencing growth and cellular organization, while necessary during developmental phases, are of diminished importance in postmitotic adult tissue.

In contrast to genes within Cluster 5, a number of genes had expression restricted to the adult stage (*e.g.*, Clusters 1 and 3). Adult abdominal tissues had significantly higher expression of many genes related to lipid and fatty acid metabolism compared with other life stages (Table 3). Insect abdominal fat bodies play a critical role in the storage and utilization of energy (reviewed in Arrese and Soulages 2010), and thus it is reasonable that lipid metabolism dominates the signal coming from abdominal overexpressed genes. Genes within Cluster 3, which are expressed in both brain and abdominal tissue of adults, are enriched for the DNA photolyase protein class. DNA photolyases are known to repair DNA damage caused by UV radiation (Sancar 1994), and may be playing a role in mitigating the effects of light exposure in *M. genalis* adults with their exceptionally sensitive eyes (Greiner *et al.* 2004).

The adult brain showed a strong signal of temporally- and spatially-restricted gene expression, with nearly 5000 genes expressed more highly in the adult brain than in any other life stage or tissue (Table 3). In both the ant *Camponotus festinatus* and in *Drosophila melanogaster*, genes highly expressed in the adult stage (including the adult brain) show a greater diversity of functional categories relative to genes more highly expressed in earlier stages (Goodisman *et al.* 2005).

The cluster of genes showing brain-restricted expression (Cluster 1, Figure 2) were also enriched for the muscarinic acetylcholine 1 and 3 signaling pathway (*P* = 0.000441). Muscarinic acetylcholine signaling has been implicated in nestmate recognition, an important feature of social behavior and potential prerequisite for social evolution (Ismail *et al.* 2008). In addition, this signaling pathway is important for foraging-dependent changes in the structure of the mushroom bodies in honey bees (Ismail *et al.* 2006). As shown in Figure 2, genes in Cluster 1 were more highly expressed in the adult brain of male individuals. Because males in this study were quite young, the inferred greater muscarinic acetylcholine receptor signaling in these individuals suggests that neuron outgrowth may be particularly enriched in early adult life of *M. genalis*, similar to what has been shown for honey bees (Fahrbach *et al.* 1998) and bumble bees (Jones *et al.* 2013). However, acetylcholine is one of the most common excitatory neurotransmitters in the insect brain (Pitman 1971; Gerschenfeld 1973), and thus could be involved in numerous other functions in adults.

### Conclusions

The ability to develop genomic resources for nonmodel organisms greatly improves our ability to use naturally occurring variation to answer important questions in evolutionary biology (Martin *et al.* 2012; Domingues *et al.* 2012). In this study, we presented a comprehensive transcriptome of development in a facultatively eusocial bee, *Megalopta genalis*, an important emerging model for understanding potential precursors to obligate eusociality among social insects. In this early broad survey of gene expression, we found a number of gene clusters with dynamic and/or temporally specific expression profiles throughout development in this bee. Many of these clusters are functionally enriched for particular classes of protein families, and thus open the door to more in-depth gene expression analyses and examinations of how the biological processes implicated here contribute to the phenotypic plasticity exhibited by *M. genalis*. Transitions between life stages of *M. genalis* display striking changes in the functional categories of expressed genes, and life stages show distinct signatures of molecular functions. These results provide a foundation for future studies of transcriptomics in *M. genalis*, as well as more in-depth analyses of gene expression plasticity in facultatively social systems.

## 
